# Novel Synthetic Approaches for Bisnaphthalimidopropyl (BNIP) Derivatives as Potential Anti-Parasitic Agents for the Treatment of Leishmaniasis

**DOI:** 10.3390/molecules24244607

**Published:** 2019-12-16

**Authors:** Elif Keskin, Mehmet Hikmet Ucisik, Bilgesu Onur Sucu, Mustafa Guzel

**Affiliations:** 1 Regenerative and Restorative Medicine Research Center (REMER), Istanbul Medipol University, 34810 Istanbul, Turkey; elkeskin@deva.com.tr (E.K.); bsucu@medipol.edu.tr (B.O.S.); 2 Department of Biomedical Engineering, School of Engineering and Natural Sciences, Istanbul Medipol University, 34810 Istanbul, Turkey; 3 Pharmacy Services, Vocational School of Health Services, Istanbul Medipol University, 34810 Istanbul, Turkey; 4 Department of Medical Pharmacology, International School of Medicine, Istanbul Medipol University, 34810 Istanbul, Turkey

**Keywords:** drug discovery for neglected rare diseases, bisnaphthalimidopropyl (BNIP) derivatives, Leishmaniasis, BNIPDaoct, BNIPDanon, novel anti-parasitic agents

## Abstract

Leishmaniasis is a neglected parasitic disease that is widely seen in more than 60 countries worldwide, including Turkey and its subcontinental region. There are several chemotherapy agents for the treatment of leishmaniasis, including pentavalent antimonials—i.e., sodium stibogluconate (Pentostan) and meglumine antimoniate (Glucantim), pentamidine, conventional amphotericin B deoxycholate, miltefosine, paramomycin (aminosidine), and liposomal amphotericin B. However, these therapies are usually unsatisfactory due to dose-limiting toxicity issues and limited efficacy. Furthermore, resistance gained by parasites endangers future success of these therapies. Addressing these issues, the development of novel drugs with high efficacy has a vital importance. Latest studies have shown that bisnaphthalimidopropyl (BNIP) derivatives display high activity against Leishmaniasis parasites by selectively targeting parasitic sirtuin proteins and interacting with DNA. Despite the promising anti-parasitic activity, the low solubility and toxicity on human macrophages are the limitations to overcome. This study describes the new synthesis strategies for existing—i.e., BNIPDaoct and BNIPDanon—and novel BNIP derivatives differing in respect of their alkyl linker chain lengths. The new synthesis approach provides certain advantages compared to its existing alternatives reported in the literature. The proposed methodology does not only decrease the number of synthesis steps and production time of BNIPDaoct and BNIPDanon, but also provides higher yields, thereby making the synthesis highly cost-effective.

## 1. Introduction

Considered as a neglected parasitic disease, Leishmaniasis is widely seen in more than 60 countries worldwide, including Turkey and its subcontinental region, affecting more than 1.5 million people with an annual death rate reaching up to 100,000 people [1,2]. It is usually transmitted by sandfly carrying protozoan kinetoplastid parasite to humans. Infective forms of this parasite are called metacyclic promastigotes that are taken up by macrophages at the vertebrate host, such as human or animal. Clinically observed human Leishmanial infections are divided into four common types, i.e., cutaneous leishmaniasis (CL), mucocutaneous leishmaniasis (MCL), diffuse cutaneous leishmaniasis (DCL), and visceral leishmaniasis (VL), where the latter is the deadliest [2]. There are several chemotherapy agents for the treatment of leishmaniasis, including pentavalent antimonials—i.e., sodium stibogluconate (Pentostan) and meglumine antimoniate (Glucantim), pentamidine, conventional amphotericin B deoxycholate, miltefosine, paramomycin (aminosidine), and liposomal amphotericin B. While the choice of chemotherapy agent varies largely depending on the type of the disease and the species, the World Health Organization (WHO) recommendations for treatment of VL—caused by *Leishmania infantum* in the Mediterranean Basin, Middle East, and Central Asia—is liposomal amphotericin B as the first-line therapy [1,2,3]. Pentavalent antimonials and amphotericin B deoxycholate are the second- and third-line choices, respectively. However, these therapies are usually unsatisfactory due to emergence of drug resistance, dose-limiting toxicity issues, and limited efficacy. Furthermore, the therapies may not always be available for undeveloped countries due to route of administration and high cost. For these reasons, orally bioavailable, more specific, and less costly new active molecules are in high demand for the control of leishmaniasis worldwide.

As part of the pursuit of new anti-parasitic therapeutics, researches are focused on selective targeting approaches, and parasitic sirtuins have recently come forward as a new drug target for leishmaniasis [4,5,6,7]. Sirtuins take a role in various biological processes, including heterochromatin, gene silencing, DNA repair, development, longevity, metabolism, adipogenesis, and apoptosis [8,9,10]. Sirtuins are evolutionarily conserved proteins present in all kingdoms of life, ranging from bacteria to humans [11]. The causative agents of infantile cutaneous and visceral leishmaniasis—i.e., *L. major* or *L. infantum*, and *L. infantum*, respectively [1]—were found to harbor a cytosolic Sir2-related protein, i.e., LmSir2rp1 and LiSir2rp1, respectively [11,12,13,14,15]. These parasitic sirtuins have emerged as novel anti-parasitic therapeutic targets, as structural studies have shown that they do differ from their human counterparts, i.e., human SIRT1 or SIRT2, by this means making them a promising drug target [5,9].

Numerous studies of Cordeira-Da-Silva and Kong Thoo Lin have recently reported that bisnaphthalimidopropyl (BNIP) derivatives, formulated by two naphthalimide groups linked with a natural polyamine chain, possess particular sirtuin inhibition and display intrinsic selectivity for parasitic sirtuin versus human SIRT1 [4,9,16,17]. BNIP derivatives displayed significant anti-proliferative effects on the life cycle of *L. infantum* and induced death of promastigotes by apoptosis [4]. The cytosolic parasite protein is an NAD^+^-dependent tubulin deacetylase and an ADP ribosyltransferase [9,18]. The mechanism of action of BNIP derivatives was shown to occur via inhibition of NAD^+^-dependent deacetylase activity of parasitic sirtuin [5,9,19]. Structural differences between the parasitic and human sirtuins are attributed as the reason for up to 17-fold selectivity [9,20].

Beside targeting the sirtuins, BNIP derivatives strongly interact with DNA—a property of naphtalimides [21]—which makes them a good drug candidate as an anticancer agent [16,22,23,24,25,26,27]. Naphthalimide planar aromatic rings of BNIP intercalate between DNA base pairs and distort the DNA backbone conformation by interfering with DNA–protein interactions [28,29]. Particular analogues may also inhibit topoisomerase II activity directly, exert post DNA damage effects on ataxia telangiectasia-mutated activated DNA damage signaling pathways, or induce lysosomal permeability [25,30,31,32]. Cell cycle arrest and apoptosis occur as a result of BNIP’s anticancer effect, as verified in different in vitro cell models [22,27,32,33,34,35].

The presence of at least one amino group at alkyl chain was found to be essential for activity of the compound [36]. Kong Thoo Lin and co-researchers have shown that the use of various natural polyamines as the linker chain does not only enhance the cytotoxicity of certain BNIP derivatives, but also improves their solubility [25,26,37]. The improved solubility is attributed to the increase in the heterogeneity of atoms in the linker chain.

In this paper, we report the new synthesis strategies for existing and new BNIP derivatives, along with certain advantages in their synthesis. The proposed methodology does not only decrease the number of synthesis steps and production time of BNIP diaminooctane (BNIPDaoct) and BNIP diaminononane (BNIPDanon), but also provides higher yields, thereby making the synthesis highly cost-effective. Our paper focuses on medicinal chemistry of the molecules. With the advantage of simplicity in synthesis, the proposed strategy may widen the studies on the use of BNIP in medicine.

## 2. Materials and Methods

### 2.1. Chemistry

All oxygen- and moisture-sensitive reactions described herein were performed in glassware that was oven dried (110 °C, 12 h) then flame dried (N_2_ stream) immediately prior to use. Reactions were conducted under an atmosphere of nitrogen, using pre-dried septa. The tips of cannulae were flame-dried under a stream of dry nitrogen gas prior to use. Most of the reaction solvents such as dimethylformamide (DMF), tetrahydrofuran (THF), dichloromethane (DCM), and acetonitrile (ACN) were purchased as sure seal dry solvents. All other reagents and solvents were purified according to standard convention. Unless indicated otherwise, silica gel chromatography (SGC) was performed on 70–240 mesh according to routine published procedures. Analytical thin-layer chromatography (TLC) was performed on glass-backed 250~plates by visualizing with anisaldehyde and phosphomolybdic acid. ^1^H Spectra were recorded at 500 MHz and ^13^C spectra at 125 MHz, both on a Bruker AM 400 instrument (Bruker, Karlsruhe, Germany). Mass spectra were recorded on a Shimadzu mass spectrometry (MS) system equipped with electron spray ionization (ESI) source operated in positive mode (Kyoto, Japan). Mass spectra were recorded on a Shimadzu mass spectrometry (MS) system. In all synthetic methods, appropriate reference methods were used. All other chemicals were purchased from Sigma Aldrich (Germany) and used as supplied. All melting points were taken on a Stuart melting point apparatus SMP30 (Staffordshire, UK).

### 2.2. Experimental

**2-(3-aminopropyl)-1H-benzo[*de*]isoquinoline-1,3(2H)-dione, (C_15_H_14_N_2_O_2_), (1a);**

Ethanol (30 mL) suspension of 1,8-naphthalic anhydride (198 mg, 1 mmol) was added on 1,3-diaminopropane (93 mg, 1.3 mmol). The reaction was stirred at 75 °C overnight. The process of reaction was controlled by TLC, and then the mixture was purified by column chromatography with ethyl acetate (EtOAc) (yield 81%) [38].

Liquid chromatography–mass spectrometry (LC–MS): [M + H]^+^ = 255.

**2-(3-hydroxypropyl)-1H-benzo[*de*]isoquinoline-1,3(2H)-dione, (C_15_H_13_NO_3_), (1b);**

1,8-naphthalic anhydride (198 mg, 1 mmol) was dissolved in dry DMF (20 mL), then 3-amino-1-propanol (75 mg, 1 mmol) and 1,8-diazabicyclo[*5.4.0*]undec-7-ene (DBU) (152 mg, 1 mmol) weer added, and stirred at 85 °C for 4 h The process of reaction was analyzed by TLC system (DCM/methanol (MeOH): 40/1). The mixture was quenched with ice-cold water and filtered to obtain pure product (yield 98%) [16].

LC–MS: [M + H]^+^ = 256.

**3-(1,3-dioxo-1H-benzo[*de*]isoquinolin-2(3H)-yl)propanoic acid, (C_15_H_11_NO_4_), (1c);**

N,N-diisopropylethylamine (DIEA) (129 mg, 1 mmol) with β-alanine (beta-alanine) (111 mg, 1.3 mmol) in ACN (30 mL) was stirred at 70 °C for 1 h. Then 1,8-naphthalic anhydride (198 mg, 1 mmol) was added and the reaction medium was stirred at 80 °C for 1 day. The process of reaction was controlled by TLC system of DCM/MeOH (20/1), and then the mixture was purified by column chromatography with TLC solution system. The product obtained as a white solid with a melting point in range of 221.8–222.5 °C (yield 72%) [39].

LC–MS: [M + H]^+^ = 284.

**Butane-1,4-diyl bis(1H-imidazole-1-carboxylate), (C_12_H_14_N_4_O_4_) (2);**

1,1′-carbonyldiimidazole (CDI) (649 mg, 4 mmol) was added to a solution of 1,4-butanediol (90 mg, 1 mmol) in THF (50 mL), and the mixture was stirred for 16 h. Stirring was continued at room temperature. The process of reaction was controlled by LC–MS. The solvent was removed under vacuum. The crude product was purified with silica gel chromatography by using EtOAc/hexanes (20/1) as eluent system. The pure product obtained as a white solid (yield 85%).

LC–MS: [M + H]^+^ = 279.

**2,2′-((octane-1,8-diylbis(azanediyl))bis(propane-3,1-diyl))bis(1H-benzo[*de*]isoquinoline-1,3(2H)-dione), (C_38_H_42_N_4_O_4_), (3);**

DBU (152 mg, 1 mmol) and 1,8-dibromoctane (272 mg, 1 mmol) were added to compound **1a** (508 mg, 2 mmol) in MeOH (60 mL), following a reflux for 4 h. The solvent was removed under vacuum. The process of this reaction was controlled by LC–MS and TLC, then purified by column chromatography with DCM/MeOH (10:1) solvent mixture (yield 60%).

LC–MS: [M + H]^+^ = 619.

^1^H NMR (500 MHz, DMSO-*d*_6_): δ = 1.25 (m, 8H, 4xCH_2_), 1.56 (m, 4H, 2xCH_2_), 2.08–1.98 (m, 4H, 2xCH_2_), 2.86 (t, *J* = 7.8 Hz, 4H, 2xNHCH_2_), 3.04–2.97 (m, 4H, 2xNHCH_2_), 4.12 (t, *J* = 6.7 Hz, 4H, 2xNCH_2_) 7.92–7.82 (m, 4H, 2xCH2), 8.51–8.38 (dd, *J* = 7.6, 5.9 Hz, 4H, ArH) ppm.

^13^C NMR (125 MHz, CDCl_3_): δ = 24.5 (CH_2_), 25.4 (CH_2_), 25.8 (CH_2_), 28.2 (CH_2_), 37.1 (CH_2_), 44.8 (CH_2_), 46.7 (CH_2_), 122.1 (C_aro_), 127.2 (C_aro_H), 127.4 (C_aro_), 130.7 (C_aro_H), 131.3 (C_aro_), 134.4 (C_aro_H), 163.7 (C=O) ppm.

**2,2′-((nonane-1,9-diylbis(azanediyl))bis(propane-3,1-diyl))bis(1H-benzo[*de*]isoquinoline-1,3(2H)-dione), (C_39_H_44_N_4_O_4_), (4);**

DIEA (259 mg, 2 mmol) and 1,9-dibromoctane (286 mg,1 mmol) were added to the mixture which included compound **1a** (508 mg, 2 mmol) in toluene/ethanol (EtOH) (1:1) (60 mL), followed by reflux for 48 h. The solvent was removed under vacuum. The process of this reaction was controlled by LC–MS and TLC. For TLC control and purification, the DCM/MeOH (7/1) system was used. Neutral alumina oxide was used to purify the product (yield 50%).

LC–MS: [M + H]^+^ = 633.

^1^H NMR (500 MHz, MeOD): δ = 1.31(m, 2H, CH_2_), 1.41 (m, 12H, 6xCH_2_), 2.20 (m, 4H, 2xCH_2_), 3.04 (m, 4H, 2xNHCH_2_), 3.13 (m, 4H, 2xNHCH_2_), 3.89 (d, *J* = 10.3 Hz, 4H, 2xNCH_2_) 7.86 (t, *J* = 7.8 Hz, 4H, ArH), 8.41 (d, *J* = 8.1 Hz, 4H, ArH), 8.60 (d, *J* = 7.1 Hz, 4H, ArH) ppm.

^13^C NMR (125 MHz, CDCl_3_): δ = 22.3 (CH_2_), 23,2 (CH_2_), 26,5 (CH_2_), 27,1 (CH_2_), 28.7 (CH_2_), 37.9 (CH_2_), 46.5 (CH_2_), 48,6 (CH_2_), 122.3 (C_aro_), 127.2 (C_aro_H), 127.3 (C_aro_), 130.7 (C_aro_H), 131.3 (C_aro_), 134.3 (C_aro_H), 163.5 (C=O) ppm.

**N^1^,N^8^-bis(3-(1,3-dioxo-1H-benzo[*de*]isoquinolin-2(3H)-yl)propyl)octanediamide, (C_38_H_38_N_4_O_6_), (5);**

While stirring at 0 °C under N_2_ atmosphere, hydroxybenzotriazole (HOBt) (135 mg, 1 mmol) was added to the solution of suberic acid (160 mg, 0.9 mmol) in 100 mL of anhydrous THF, and stirred for 10 min. Then, *N,N*′-dicyclohexylcarbodiimide (DCC) (206 mg, 1 mmol) was added to the reaction mixture. After 35 min, compound **1a** (508 mg, 2 mmol) was added to the reaction mixture and stirred at room temperature for 24 h. The formed precipitate of DCU was removed by filtration, and the filtrate was evaporated under vacuum. The residue was dissolved in 50 mL EtOAc. The formed solution was washed successively with saturated aqueous solution of sodium bicarbonate (NaHCO_3_) (30 mL × 3), 5% aqueous solution of potassium bisulfate (KHSO_4_) (30 mL × 3), and saturated aqueous solution of sodium chloride (NaCl) (30 mL × 3), and then dried with anhydrous sodium sulfate (Na_2_SO_4_) [40]. After filtration, the filtrate was evaporated under vacuum to give the title compound. EtOAc/ n-hexane (1/ 20) solvent system was used for the purification (yield 61%). 

LC–MS: [M + H]^+^ = 647.

^1^H NMR (500 MHz, CD_3_Cl), δ; 1.35 (s, 4H, 2xCH_2_), 1.71–1.60 (m, 4H, 2xCH_2_), 1.88 (t, *J* = 6.2 Hz, 4H, 2xCH_2_), 2.21 (t, *J* = 7.6 Hz, 4H 2xNCH_2_), 3.20 (q, *J* = 6.1 Hz, 4H, 2xNHCH_2_), 4.18 (t, *J* = 6.3 Hz, 4H, 2xO = CCH_2_), 6.46 (t, *J* = 6.2 Hz, 2H, 2xNH), 7.69 (t, J = 8.1 Hz, 4H, ArH), 8.15 (d, J = 8.2 Hz, 4H, ArH), 8.52 (d, J = 7.2 Hz, 4H, ArH) ppm.

***N*,*N*′-(octane-1,8-diyl)bis(3-(1,3-dioxo-1H-benzo[*de*]isoquinolin-2(3H)-yl)propanamide), (C_38_H_38_N_4_O_6_), (6);**

While stirring at 0 °C under N_2_ atmosphere, HOBt (135 mg, 1 mmol) was added to the anhydrous THF solution (100 mL) of compound **1c** (260 mg, 0.9 mmol) for 10 min. Then, DCC (206 mg, 1 mmol) was added to the reaction mixture. After 35 min, 1–8 diaminooctane (544 mg, 2 mmol) was added and the reaction mixture was stirred at room temperature for 24 h. The formed precipitate of DCU was removed by filtration. The filtrate was evaporated under vacuum. The residue was dissolved in 50 mL of EtOAc. The formed solution was washed successively with saturated aqueous solution of NaHCO_3_ (30 mL × 3), 5% aqueous solution of KHSO_4_ (30 mL × 3), and saturated aqueous solution of NaCl (30 mL × 3), then was dried with anhydrous Na_2_SO_4_ [40]. After filtration, the filtrate was evaporated under vacuum to give the title compound. EtOAc/ n-hexane (1/ 20) solvent system was used for the purification (yield 57%).

LC–MS: [M + H]^+^ = 647.

^1^H NMR (500 MHz, CDCl_3_): δ = 0.89–0.74 (m, 2H, CH_2_), 1.30–1.09 (m, 4H, CH_2_), 1.50 (brs, 4H, CH_2_), 2.81–2.60 (m, 4H, NHCH_2_), 3.63 (brs, 6H, NCH_2_), 4.52–4.31 (m, 4H, OCH_2_), 7.69 (dd, *J* = 8.1, 7.4 Hz, 4H, ArH), 8.16 (dd, *J* = 8.4, 0.9 Hz, 4H, ArH), 8.54 (dd, *J* = 7.3, 1.1 Hz, 4H, ArH) ppm.

^13^C NMR (125 MHz, CDCl_3_): δ = 32.5 (CH_2_), 36.1 (CH_2_), 122.5 (C_aro_), 126.9 (C_aro_H), 128.2 (C_aro_), 131.3 (C_aro_H), 131.6 (C_aro_), 134.1 (C_aro_H), 164 (C=O), 171.7 (C=O) ppm.

**Butane-1,4-diyl bis((3-(1,3-dioxo-1H-benzo[*de*]isoquinolin-2(3H)-yl)propyl)carbamate), (C_36_H_34_N_4_O_8_), (7);**

Compound **2** (1.2 g, 4.4 mmol) and DIEA (2.1 g, 8 mmol) were added to a solution of **1a** (254 mg, 1 mmol) in THF (40 mL), and the mixture was stirred for 16 h. Stirring was continued at room temperature. The process of reaction was monitored and controlled by TLC and LC–MS. The progress of the reaction and the purification of the product were carried out by either precipitation with MeOH or gel silica and column chromatography (DCM/ MeOH, 50/1). The pure product obtained as a white solid (yield 74%).

LC–MS: [M + H]^+^ = 651.

^1^H NMR (500 MHz, CDCl_3_): δ = 1.64 (d, *J* = 12.8 Hz, 4H, 2xCH_2_), 1.98–1.79 (t, J = 6.5 Hz, 4H, 2xCH2), 3.15 (q, *J* = 6.3 Hz, 4H, 2xCH_2_), 4.03 (m, 4H, 2xCH_2_), 4.21 (t, *J* = 6.4 Hz, 4H 2xCH_2_), 5.50 (brd, *J* = 6.3 Hz, 2xNH), 7.69 (t, *J* = 7.7 Hz, 4H, ArH), 8.15 (d, *J* = 8.2 Hz, 4H, ArH), 8.53 (d, *J* = 7.3 Hz, 4H, ArH) ppm.

^13^C NMR (125 MHz, CDCl_3_): δ = 25.7 (CH_2_), 28.3 (CH_2_), 37.5 (CH_2_), 37.8 (CH_2_), 64.4 (O-CH_2_), 122.4 (C_aro_), 127 (C_aro_H), 128.1 (C_aro_), 131.4 (C_aro_H), 131.3 (C_aro_), 134.1 (C_aro_H), 156.7 (C=O), 164.5 (C=O) ppm.

**Bis(3-(1,3-dioxo-1H-benzo[*de*]isoquinolin-2(3H)-yl)propyl) butane-1,4-diyldicarbamate, (C_36_H_34_N_4_O_8_), (8);**

Compound **1b** (510 mg, 2 mmol) was added to a solution of cesium carbonate (Cs_2_CO_3_) (2.6 g, 8 mmol) in anhydrous THF (80 mL), and the mixture was stirred for 1 h. Then, 1,4-diisocyanatebutane (140 mg, 1 mmol) was added to reaction mixture. Stirring was continued at room temperature for 17 h. All procedures were carried out under N_2_ atmosphere. The process of reaction was controlled by LC–MS. The EtOAc/n-hexane (4/1) solvent system was used for the reaction progress and purification by column chromatography. MP: 205–206 °C (yield 70%).

LC–MS: [M + H]^+^ = 651.

^1^H NMR (500 MHz, CDCl_3_): δ = 1.55–1.36 (m, 4H, CH_2_), 2.03 (d, *J* = 8.6 Hz, 4H, NHCH_2_), 3.27–3.01 (m, 4H, NCH_2_), 4.33–4.04 (m, 4H, OCH_2_), 7.67 (dd, *J* = 16.4, 8.4 Hz, 4H, ArH), 8.12 (d, *J* = 8.2 Hz, 4H, ArH), 8.50 (d, *J* = 7.1 Hz, 4H, ArH) ppm.

## 3. Results and Discussion

**Figure 1:**
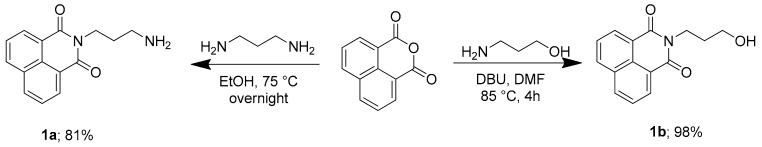
Synthesis schemes of initial intermediate compounds: Compounds **1a** and **1b**.

**Figure 2:**
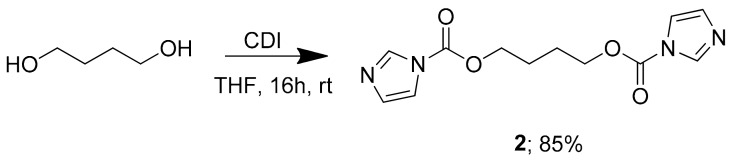
Synthesis scheme of compound **2.**

This study reports the synthesis of existing (i.e., compounds **3** and **4**) and novel BNIP analogs (i.e., compounds **5, 6, 7** and **8**) that may possess potential anti-parasitic activity. BNIPDaoct (compound **3**) and BNIPDanon (compound **4**) that were developed by Lin and Pavlov [37] were taken as the starting point of the study. The new derivatives differ from these in respect of their alkyl linker chains. Accordingly, the intermediate compounds, compounds **1a** and **1b**, were synthesized as illustrated in Figure 1; and compound **2** was synthesized in the CDI and butanediol in THF as illustrated by Figure 2.

**Figure 3:**
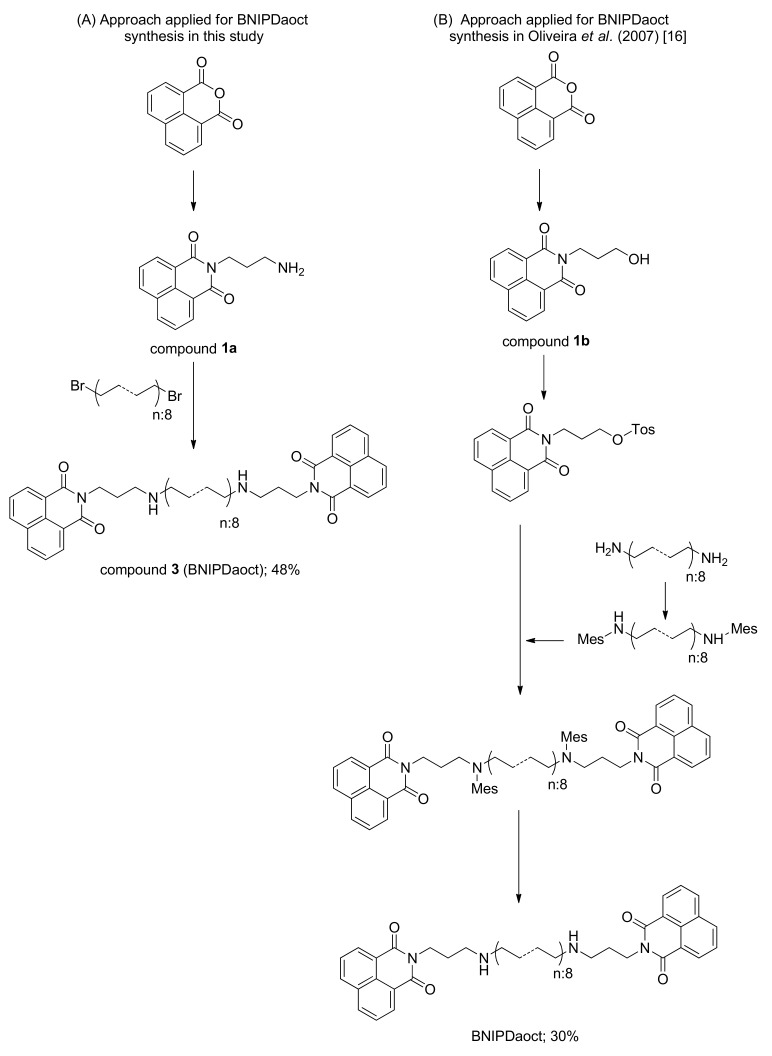
Schematic illustrations of approaches applied for BNIPDaoct synthesis (**A**) in this study and (**B**) in Oliveira et al. [16].

Different from the synthesis procedure described in detail in Oliveira et al. [16], where a five-step synthesis was designated, BNIPDaoct was synthesized in our approach within two steps (Figure 3). According to the previous approach (Figure 3B), first the naphthalimidopropanol compound was obtained by reaction of 1,8-naphthalic anhydride and 3-aminopropanol in the presence of DBU base and DMF solvent, affording the production of target compound, i.e., compound 1b, with 95% yield [16]. This intermediate was then reacted with *p*-toluenesulfonyl chloride for a tosylation reaction in pyridine solvent at 0 °C temperature to afford *O*-tosylpropylnaphthalimide compound with 53% yield. The 1,8-diaminooctane compound was activated by treatment with mesitylene chloride at room temperature in the pyridine solvent to give N^1^,N^8^-dimesityloctane compound in 70% yield (Figure 3B). Dimesitylated intermediate was coupled with *O*-tosylpropylnaphthalimide compound in the next step. The synthesized N^1^,N^8^-dimesityloctane, and the *O*-tosylpropylnaphthalimide were used as starting material of the N-alkylation reaction. BNIPDaoct compound was then obtained with 85% yield by deprotection reaction, where anhydrous dichloromethane solvent and hydrobromic acid/glacial acetic acid reagent were used to remove mesyl protecting groups in the backbone of the compound, and thereby fully protected polyamine derivatives were obtained. With each single-reaction yield given, the five-step synthesis of BNIPDaoct in Oliveira et al. [16] occurs within an overall yield of 30%.

In contrast to this previous approach, the synthesis of BNIPDaoct (compound **3**) was achieved within two steps through our new approach. Accordingly, the substitution reaction was first carried out as indicated in Figure 3A. The substitution reaction between 1,8-naphthalic anhydride and 1,3-diaminopropane at 75 °C in ethanol as solvent provided the desired intermediate compound **1a** (Naphthalimidopropanamine, [2-(3-aminopropyl)-1H-benzo[*de*]isoquinoline-1,3(2H)-dione]) in 81% yield. In the second and final step, compound 1a and 1,8-dibromoctane were let into reaction in the presence of DBU base in MeOH solvent to produce BNIPDaoct (compound **3**) with 60% yield (Figure 3A). Two-step BNIPDaoct synthesis occurred with an overall yield of 48%, which is better than the yield achieved in Oliveira et al. [16]. 

The lack of three additional chemical steps and higher yield clearly provides cost effectiveness for the synthesis of not only BNIPDaoct, but also other BNIP derivatives such as BNIPDanon (compound **4**) and compounds **5** and **7**.

As an observation, it is also important to state that, for the synthesis of compound **1a**, at first, we attempted the reaction conditions with organic solvent such as dry DMF and organic bases such as DBU and DIEA, but this synthesis method was later changed due to extremely low yields. Therefore, adaptation to the outlined approach not only shortened the synthesis, but also overcame the challenges in the synthesis of BNIP analogs.

**Figure 4:**
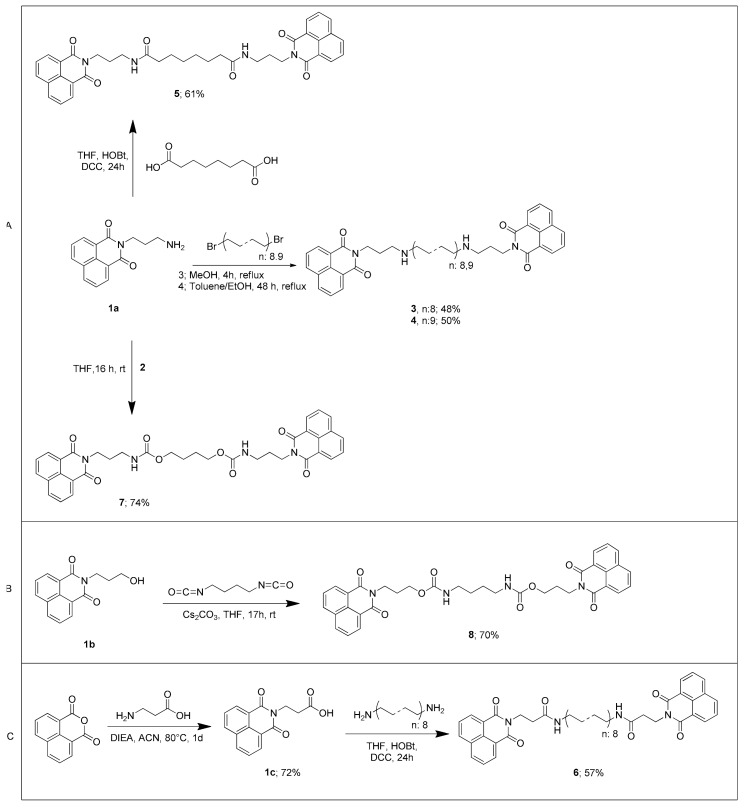
Synthesis schemes for (**A**) compounds **3** (BNIPDaoct), **4** (BNIPDanon), **5**, and **7**; (**B**) compound **8**; and (**C**) compound **6**.

^13^C NMR analysis of compound **3** (BNIPDaoct) and compound **4** (BNIPDanon) is presented in Table 1. Compound **4** (BNIPDanon) is the homologated version of compound **3** (BNIPDaoct) that has additional CH_2_ moiety in the linker portion. Thus, again applying the two-step chemical approach described in Figure 3A, BNIPDanon (compound **4**) was successfully synthesized with 50% yield (Figure 4A).

Compound **5** was obtained as a result of the amide reaction of the amine group of the first step product, compound **1a**, with subaric acid (Figure 4A). Compound **6** was obtained by amide coupling procedure with the amine groups at both ends of the diamine using 1–8 diaminooctane in the amide reaction, where compound **1c**, 3-(1,3-dioxo-1H-benzo[*de*]isoquinolin-2(3H)-il)propanoic acid (C_15_H_11_NO_4_), was used as the starting material (Figure 4C).

For the synthesis of compound **8**, firstly we attempted the conditions same as in the synthesis of compound **7** prior to carbonation of compound **1b** with CDI, followed by the reaction of 1,4-diaminobutane. However, this method was abandoned due to long four-step synthesis and low yields in the reactions. For that reason, compound **1b** was synthesized initially as described in Figure 1, which was then followed by a reaction with 1,4-diisocyanatobutane, affording compound **8** with 70% yield (Figure 4B).

Although the synthetic feasibility of these novel BNIP analogs looked very straightforward, several challenges were faced during the synthesis, mainly due to the highly hydrophobic characters of BNIPs and their initial or intermediate compounds. These challenges were overcome through the innovated synthesis approach introduced in this study (Figure 3A). Linker modifications are seen as the prominent solution to enhance the solubility of BNIP analogs, which may potentially improve the activity of the compounds. Herein, this study reports a new and more feasible chemistry approach for the synthesis of both existing and novel BNIP analogs containing various substituents as drug-like moieties. While the yields of the production seem to vary depending on the structure of the analogs, the synthetic feasibility of these molecules evidently provides insights about the novel two-step synthesis approach.

## 4. Conclusions

In conclusion, this study introduces a concise and flexible approach for the assembly of a wide variety of BNIP-scaffold analogs. Novel BNIP analogs—including compounds **5**–**8**—are designed in order to improve the solubility and the activity of the existing molecules, i.e., BNIPDaoct (compound **3**) and BNIPDanon (compound **4**). Since 8 or 9 carbon linkers were the optimum linkers based on the preliminary assay results against *L. infantum* promastigotes [16], linker modification analogs of these BNIP molecules were firstly designed. Accordingly, functional groups such as amides, ureas, carbametes, reverse amides, and reverse carbametes were added to the carbon chain backbone of BNIPDaoct and BNIPDanon. Although the chemistry reported by Kong Thoo Lin and Pavlov (2000) looked very straightforward [37], some serious issues related with low yield and solubility obliged alteration of the synthesis strategy during the study, which rewardingly resulted in development of a more cost-effective and faster route for the synthesis of BNIP molecules, as presented for the first time in this study. Two-step synthesis of BNIP derivatives stands as an alternative approach to the synthesis procedure reported in the literature [16,37].

A salient feature of the presented approach is avoidance of longer steps, as well as higher costs, for synthesis. This route is complementary to known methodologies [16,37] and should be applicable for the preparation of many interesting compounds hosting a substituted with different linker motifs. Thus, modifications to the linker chain, as well as on the aryl groups in the BNIP derivatives, might show some improvements in its intrinsic biological activities against Leishmaniasis, as well as cancer, which will be the topic of our future studies. The cellular activity of newly synthesized BNIP derivatives is currently being investigated on *L. infantum* parasites and their results will be reported separately in the future.

## Figures and Tables

**Table 1:** ^13^C NMR analysis of compound 3 (BNIPDaoct) and compound 4 (BNIPDanon)**.**

	C_1_	C_2_	C_3_	C_4_	C_5_	C_6_	C_7_	C_8_	C_9_	C_10_	C_11_	C_12_	C_13_	C_14_	C_15_	
**Compound 3 (BNIPDaoct)**	163.7	131.3	134.4	130.7	127.7	122.1	127.4	46.7	28.2	44.8	37.1	25.8	25.4	24.5	-	**ppm**
**Compound 4 (BNIPDanon)**	163.5	131.2	134.3	130.7	127.2	122.3	127.3	48.6	28.7	46.5	37.9	27.1	26.5	23.2	22.3	
